# Using an External Exposome Framework to Examine Pregnancy-Related Morbidities and Mortalities: Implications for Health Disparities Research

**DOI:** 10.3390/ijerph13010013

**Published:** 2015-12-22

**Authors:** Tonny J. Oyana, Patricia Matthews-Juarez, Stephania A. Cormier, Xiaoran Xu, Paul D. Juarez

**Affiliations:** 1 Research Center on Health Disparities, Equity & the Exposome, Department of Preventive Medicine, University of Tennessee Health Science Center, Memphis, TN 38163, USA; 2 Pediatrics, Infectious Disease and Microbiology, Immunology & Biochemistry, University of Tennessee Health Science Center, Le Bonheur Children’s Medical Center, Memphis, TN 36163, USA; pmatthews-juarez@mmc.edu (P.M.-J.); scormier@uthsc.edu (S.A.C.); xxu24@uthsc.edu (X.X.); pjuarez@mmc.edu (P.D.J.); 3 Department of Family and Community Medicine, Meharry Medical College, Nashville, TN 37208

**Keywords:** external exposome, health disparities, pregnancy outcomes, maternal mortality, infant mortality, premature birth, low birthweight, geographic information systems, discrete Poisson model

## Abstract

*Objective*: We have conducted a study to assess the role of environment on the burden of maternal morbidities and mortalities among women using an external exposome approach for the purpose of developing targeted public health interventions to decrease disparities. *Methods*: We identified counties in the 48 contiguous USA where observed low birthweight (LBW) rates were higher than expected during a five-year study period. The identification was conducted using a retrospective space-time analysis scan for statistically significant clusters with high or low rates by a Discrete Poisson Model. *Results*: We observed statistically significant associations of LBW rate with a set of predictive variables. However, in one of the two spatiotemporal models we discovered LBW to be associated with five predictive variables (teen birth rate, adult obesity, uninsured adults, physically unhealthy days, and percent of adults who smoke) in two counties situated in Alabama after adjusting for location changes. Counties with higher than expected LBW rates were similarly associated with two environmental variables (ozone and fine particulate matter). *Conclusions*: The county-level predictive measures of LBW offer new insights into spatiotemporal patterns relative to key contributory factors. An external framework provides a promising place-based approach for identifying “hotspots” with implications for designing targeted interventions and control measures to reduce and eliminate health disparities.

## 1. Introduction

The Research Center on Health Disparities, Equity, and the Exposome employs a transdisciplinary, external exposome framework. This framework integrates diverse data for exposure assessment and uses sophisticated tools and methods, such as spatial analytics, Geographic Information Systems (GIS), spatiotemporal and computational models to identify health disparities “hotspots” among and within subpopulations in order to design place-based, public health interventions. Geographically-targeted interventions to break the chain of disparity in a subpopulation and the indicated geographic region are needed to support public health decision-making and policies. In previous work [1], we applied the external exposome framework to a subset of a previously established multidimensional, spatiotemporal data repository of county-level health, socioeconomic, and environmental measures obtained from publicly available sources including the Centers for Disease Control and Prevention (CDC), Environmental Protection Agency (EPA), Census Bureau, Health Resources and Services Administration (HRSA), *etc*.

The external exposome approach builds on the work of Wild [2,3], who first coined the term “exposome” as a measure of the totality of life-long environmental exposures on health. It also addresses the need identified by Payne-Sturges and Gee [4] for a model to test empirically the interrelations between social, physical, and built environments and health disparities. In this current effort, we applied an external exposome framework, geographic information systems, and spatiotemporal models to study the interrelationships between multiple health and environmental dimensions and several health outcomes among women.

### 1.1. Background

Low income, African American women who do not complete high school, and present later in their pregnancy for prenatal care, have a higher than average prevalence of unintended pregnancies and higher than average rates of pregnancy-related morbidities and mortalities [5]. They also have higher prevalence of overweight/obesity, alcohol use, exposure to second hand smoke before and during pregnancy compared to non-Hispanic White women, lack preconception care, have limited access to other healthy reproductive and sexual practices or are disproportionately affected by chronic diseases that negatively impact pregnancy outcomes [6,7,8,9,10]. Over the last 50 years, the risk of death from pregnancy complications for African American women was four times higher than for White women [11]. From 1990 to 2008, the maternal mortality rate among African American women in the United States nearly doubled [12]. Between 2006 and 2009, maternal mortality rates by race were 11.7 deaths per 100,000 live births for White women and 35.6 deaths per 100,000 live births for black women. Studies suggest that between 20%–50% of these maternal deaths were preventable [13]. The top five causes of maternal mortality were embolism, hemorrhage, preeclampsia and eclampsia, infection, and cardiomyopathy [14]. Tucker *et al.* [11] examined the prevalence of common pregnancy complications and associated fatality rates. African American women also were found more likely to have preexisting medical conditions (*i.e.*, hypertension, diabetes, asthma, connective tissue disease, human immunodeficiency virus, genitourinary infections, and periodontal disease), have unintended pregnancies, and to be publically insured and/or Medicaid eligible due to pregnancy [15]. While the number of deaths from hemorrhage has declined in recent years, deaths from pulmonary embolus and hypertension have not [16].

### 1.2. Infant Mortality, Premature Birth, Low Birthweight, and Maternal Morbidities

In 2007, the infant mortality rate for African American women nationally was 2.4 times the rate for White women [17]. The leading cause of infant death among African American women was short gestation and/or low birthweight (LBW) [17]. Increased risk of infant mortality has been associated with lower socioeconomic status, lower educational attainment, decreased access and utilization of prenatal care, maternal stress, infections, and experiences of racism [18]. These factors, however, only partially explain racial/ethnic disparities in infant mortality [19].

The risk of premature birth is particularly heightened among women with a previous premature birth, multiple pregnancy, cervical or uterine abnormalities, and certain lifestyle factors. The number of African American women with LBW infants (less than 2500 g) is nearly two-fold compared to white women. Recent reports link LBW with a number of adverse health outcomes later on in children’s life, including low cognitive performance, learning disabilities, lower height-for-age, and increased risks of mortality and/or morbidity [20,21,22,23,24]. Numerous epidemiological studies have identified key contributing factors, such as maternal age, parity, weight gain, smoking, premature birth (less than 37 weeks gestation) as being responsible for LBW outcomes [21,23,25].

While the increasing rate of pregnancy-related mortality is alarming, severe maternal morbidities are 50 times more common [17]. Rates of racial and ethnic disparities in maternal morbidities were three to four times higher for African American women than for White, Hispanic and Asian American women [18]. Known risk factors for severe maternal morbidities included increased maternal age, self-pay or Medicaid coverage, low socioeconomic status, and preexisting chronic medical conditions. African American women who become eligible for Medicaid due to pregnancy often have delays in seeking prenatal care or have difficulty finding providers who accept Medicaid. It is well documented that women who do not receive or begin prenatal care after the first trimester have an increased risk of pregnancy-related complications compared to women who receive timely prenatal care [12]. If an African American woman is uninsured or enters pregnancy with chronic medical conditions, the risk of pregnancy-associated morbidity is increased.

The majority of women of reproductive age in the United States are overweight or obese [19], with African American women having an increased risk of obesity [15]. Obesity is associated with a variety of adverse pregnancy complications including cesarean delivery, gestational diabetes, and preeclampsia [19]. Interestingly, low socioeconomic status (SES) is not associated with increased maternal morbidities among African American women. These risk factors only partially explain the disparities in maternal morbidities in African American women [18]. When African American and White women present with the same comorbidities (*i.e.*, hypertension, diabetes, asthma, human immunodeficiency virus), African American women fare worse in pregnancy than White women [15]. Bruce *et al.* [20] found that the five most common maternal complications were urinary tract infections, anemia, mental health conditions, pelvic and perineal complications, and obstetrical infections. Compared to other racial and ethnic groups, African American women have a greater proportion of pregnancies that were associated with at least one of these complications.

The underlying causes of the disparities in pregnancy-related morbidities and mortalities are complex and have proven difficult to unravel. While many individual risk factors associated with poor pregnancy-related outcomes are known, the complex spatial/temporal nature of the relationships between health outcomes, environmental exposures and population level disparities are not yet well understood. Environmental factors that have been previously identified include characteristics about health care providers, access to a timely, culturally competent prenatal care, and exposures found in the natural, built, social, and policy domains [26]. Though significant progress has been made, some gaps still persist in pregnancy-related morbidities and mortalities literature partly because many of the factors are interwoven, complex, and poorly understood. Besides, most studies on pregnancy-related morbidities and mortalities have relied heavily on hospital administrative data and do not assess clinical, environmental exposures, or systems level factors or account for complications that are treated in outpatient settings [27,28].

**Figure 1:**
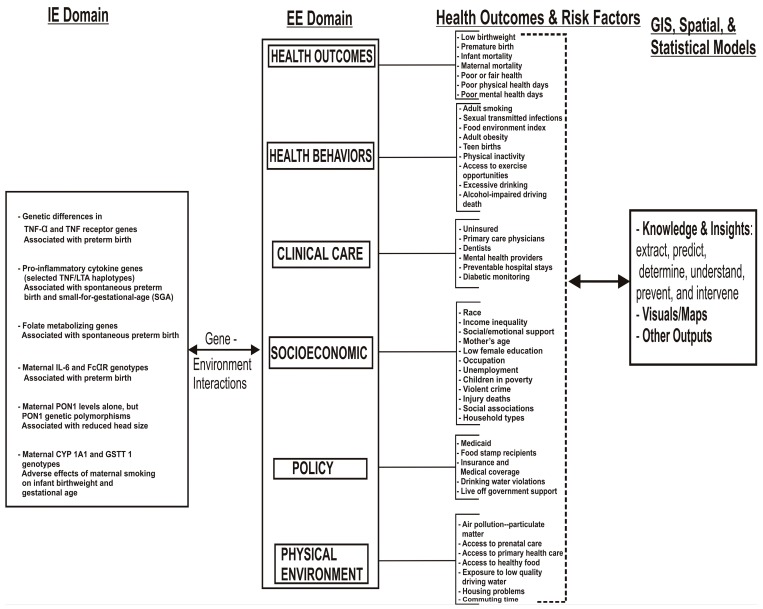
A conceptual framework for understanding adverse birth outcomes synthesized from over 50 research articles. IE refers to internal exposome while EE is the external exposome.

Figure 1 provides a conceptual exposome framework for understanding adverse birth outcomes. This framework consists of the external and internal exposome domains, health outcomes and risk factors, and analytical tools, methods, and strategic goals for revealing novel patterns and insights. Although a number of recent studies have been conducted in this emerging exposome area, there is still little information on geographically-integrated health measures. In this study, we have reviewed genetic factors that are specific to the internal exposome domain or are reported to be associated with preterm birth, reduced head size, infant birthweight, and premature birth. Non-genetic factors that make up the external exposome domain and are specific to this application include, health outcomes, health behaviors, clinical care, socioeconomic, policy and programs, and the physical environment.

The framework, which combines exposome, the use of GIS and spatial analysis, spatiotemporal models, computational and traditional statistical analytics, is applied to study the complex relationships of LBW across U.S. counties. The framework addresses the total life span of individuals in relation to their exposure and offers a better way to examine contributory factors, and thus may provide new insights. The objective of this paper is to assess geographic variation and role of environment on the burden of LBW among women for the purpose of developing targeted public health interventions to decrease disparities. We used spatiotemporal models to identify counties in the 48 contiguous USA where observed LBW rates were higher than expected during a five-year study period. This was conducted using a retrospective space-time analysis scan for statistically significant clusters in counties with high or low rates by a Discrete Poisson Model. The findings from this study could be used to develop targeted, public health interventions to decrease disparities. The findings can also serve as a basis for designing multilevel culturally-situated interventions to address ethnic disparities. Interventions that incorporate geographic parameters provide a more promising approach and will yield geographically targeted, optimal control and preventive strategies to reduce ethnic disparities in pregnancy-related morbidities and mortalities.

## 2. Methods

### Exploring Low Birthweight Variations Using Powerful Spatiotemporal Models

For this study, we used the 2010–2014 County Health National Data compiled by the Robert Wood Johnson Foundation and the University of Wisconsin Population Health Institute (http://www.countyhealthrankings.org/). The two institutions have developed a robust list of health measures at county-level since 2010. Most of their health variables/measures are derived from the Behavioral Risk Factors Surveillance System survey and U.S. Census Bureau. Air quality monitor data comes from the Public Health Air Surveillance Evaluation project. This project was a collaborative effort between the CDC and EPA, which yielded a robust spatiotemporal model for estimating fine particulate matter concentrations throughout the year. The downloaded datasets were processed using Microsoft Excel (Microsoft Inc., Redmond, WA, USA) and ArcGIS 10.2.2 (ESRI Inc., Redlands, CA, USA). Descriptive and confirmatory statistical analyses were done using the same software packages. But the ordinary least square (OLS) models were generated using IBM SPSS Version 22 (SPSS Inc.; IBM Corp, Armonk, NY, USA) and the spatiotemporal models were created using SaTScan (developed jointly by Kulldorff M., Boston, Massachusetts and Information Management Services, Inc., Silver Spring, MD, USA). The best OLS and spatiotemporal regression models were created after conducting a comprehensive screening and exploratory analysis of 47 variables of interest (see Table 1). All diagnostic statistics were analyzed for all the models, including model fitness and collinearity results.

Modeling the relationship between low birthweight and contributory factors followed a two-step process. The first step was the development of six OLS models. In the second step, we used Kulldorff’s retrospective Space-Time Statistics to determine whether high or low rates of LBW were randomly distributed in the United States over space and time. For all of these models, we assumed that LBW reported at county-level in the 48 contiguous USA (3109 counties) was dependent on the 47 predictor variables during the study period.

We constructed five regression models for each year (2010–2014) to explain the link between LBW and predictor variables. Each of the reduced models had between 12 and 17 predictor variables that were influential. Consequently, 17 predictor variables were used to create the overall regression model for LBW for the entire period. Upon compilation and synthesis of the model results, we determined that only nine influential predictors consistently explained LBW outcomes in all five of the models that were created. Using this new knowledge, we developed two retrospective spatiotemporal models for LBW outcomes.

## 3. Results

Table 2 gives the OLS model outputs for LBW and the most influential predictors, with some being statistically significant at each time point. The best overall model yielded an R^2^ of 0.705 based on backward stepwise regression criteria. The R^2^ results for each of the years were quite close to one another: the 2010 model was 0.650, the 2011 model was 0.659, the 2012 model was 0.668, the 2013 model was 0.647, and the 2014 model was 0.639. In addition, we observed a general improvement in the R^2^ value and fitness statistics for the overall model. Nine influential predictors were temporally consistent and able to explain LBW variations in all the five models. These predictors included ambulatory care sensitive conditions discharge rate, teen births rate, percent of adults 18–64 without insurance, percent of adults that report BMI ≥ 30, mentally unhealthy days per month, age-adjusted years of potential life lost rate, percent of white, percent of Native Americans, and percent of Hawaiian or Pacific Islander.

**Table 1:** A list of variables used in the exploratory regression model.

Measure	Description	Data Source	Years of Data
HEALTH OUTCOMES			
Premature death	Years of potential life lost before age 75 per 100,000 (age-adjusted)	National Center for Health Statistics—Mortality files	2010–2012
Poor or fair health	% of adults that report fair or poor health (age-adjusted)	Behavioral Risk Factor Surveillance System	2006–2012
Poor physical health days	Average # of reported physically unhealthy days per month	Behavioral Risk Factor Surveillance System	2006–2012
Poor mental health days	Average # of reported mentally unhealthy days per month	Behavioral Risk Factor Surveillance System	2006–2012
Low birthweight	% of births with low birth weight (<2500 g)	Behavioral Risk Factor Surveillance System	2006–2012
HEALTH FACTORS			
HEALTH BEHAVIORS			
Adult smoking	% of adults that reported currently smoking	Behavioral Risk Factor Surveillance System	2006–2012
Adult obesity	% of adults that report BMI ≥ 30	CDC Diabetes Interactive Atlas	2011
Food environment index	Indicator of access to healthy foods—0 is worst, 10 is best	USDA Food Environment Atlas, Map the Meal Gap	2012
Physical inactivity	% of adults that report no leisure-time physical activity	CDC Diabetes Interactive Atlas	2011
Access to exercise opportunities	% of the population with access to places for physical activity	Business Analyst, Delorme map data, ESRI & US Census Tigerline Files	2010 & 2013
Access to recreational facilities	% of recreational facility access per 100,000	USDA Food Environment Atlas, Map the Meal Gap	2008
Limited access to healthy foods	% of people with limited access to health foods	USDA Food Environment Atlas, Map the Meal Gap	2008
Fast food restaurants	% of restaurants that are fast food restaurants	USDA Food Environment Atlas, Map the Meal Gap	2008
Binge drinking	% of adults that report binge drinking	Behavioral Risk Factor Surveillance System	2006–2012
Alcohol-impaired driving deaths	% of driving deaths with alcohol involvement	Fatality Analysis Reporting System	2009–2013
Sexually transmitted infections	# of Chlamydia cases per 100,000	National Center for HIV/AIDS, Viral Hepatitis, STD, and TB Prevention	2012
Teen birth rate	# of births per 1000 female population ages 15–19	National Center for Health Statistics—Mortality files	2006–2012
CLINICAL CARE			
Uninsured adults	% of adults ages 18–64 without insurance	Small Area Health Insurance Estimates	2012
Primary care provider rate	# of primary care physicians per 100,000	Area Health Resource File/American Medical Association	2012
Mental health providers	Ratio of population to mental health providers	CMS, National Provider Identification File	2014
Preventable hospital stays	# of hospital stays for ambulatory-sensitive conditions per 1000 Medicare enrollees	Dartmouth Atlas of Health Care	2012
Diabetic screening	% of Diabetic Medicare enrollees receiving HbA1c test	Dartmouth Atlas of Health Care	2012
Mammography screening	% of female Medicare enrollees ages 67–69 having at least 1 mammogram in 2 years	Dartmouth Atlas of Health Care	2012
SOCIAL AND ECONOMIC FACTORS			
High school graduation	Calculated averaged freshman graduation rate	data.gov, supplemented w/National Center for Education Statistics	2011–2012
High school graduation	Graduation rate (Cohort or Averaged Freshman)	data.gov, supplemented w/National Center for Education Statistics	2011–2013
College degrees	% of adults ages 25–44 with some post-secondary education	American Community Survey	2009–2013
Unemployment	% of population age 16+ unemployed and looking for work	Bureau of Labor Statistics	2013
Children in poverty	% of children under age 18 living in poverty	Small Area Income and Poverty Estimates	2013
Income inequality	Gini coefficient of household income inequality	American Community Survey	2009–2013
Inadequate social support	% of adults that report not getting social/emotional support	Behavioral Risk Factor Surveillance System	2006–2012
Single-parent households	% of households that are single-parent households	American Community Survey	2009–2014
Violent crime rate	# of violent crimes per 100,000	Uniform Crime Reporting—FBI	2010–2012
Homicide rate	# of homicides per 100,000 (age-adjusted)	Uniform Crime Reporting—FBI	2000–2006
Injury deaths	# of deaths due to injury per 100,000	CDC WONDER mortality data	2008–2012
Age	Different Mother Age intervals	U.S Census Bureau	2010
Race	Different Racial/Ethnic groups	U.S Census Bureau	2010
PHYSICAL ENVIRONMENT			
Air pollution-particulate matter days	# of days that air quality was unhealthy due to fine particulate matter	CDC WONDER environmental data	2011
Air pollution-ozone days	# of days that air quality was unhealthy due to ozone	CDC WONDER environmental data	2011
Daily fine particulate matter	Average daily PM2.5	CDC WONDER environmental data	2011
Drinking water safety	% of population in violations	Safe Drinking Water Information System	2013–2014
Severe housing problems	Calculated averaged freshman graduation rate	Comprehensive Housing Affordability Strategy (CHAS) data	2007–2011
Driving alone to work	% of people who drive alone to work	American Community Survey	2009–2013
Long commute-driving alone	Among workers who commute in their car alone, the percentage that commute more than 30 min	American Community Survey	2009–2013

Data Source: County Health National Data available at http://www.countyhealthrankings.org/

**Table 2:** Estimates of multivariate linear regression model for low birthweight. Nine influential predictors were consistently present in all of the six models and were able to explain low birthweight outcomes in 2010, 2011, 2012, 2013, and 2014.

Variable	2010	2011	2012	2013	2014	Overall Model 2010–2014
	Coefficient	Coefficient	Coefficient	Coefficient	Coefficient	
Percent of low birthweight	8.255	6.115	10.28	12.009	8.974	9.745
	(16.702) **	(9.753) **	(17.224) **	(18.45) **	(21.42) **	(23.107) **
Ambulatory care sensitive conditions discharge rate	0.004	0.004	0.006	0.004	0.009	0.005
	(3.871) **	(2.746) *	(5.832) **	(3.333) **	(7.322) **	(4.867) **
Teen births rate	0.041	0.042	0.030	0.039	0.026	0.029
	(17.743) **	(13.496) **	(13.007) **	(14.556) **	(9.843) **	(11.877) **
Percent of adults 18–64 without insurance	−0.071	−0.044	−0.086	−0.103	−0.074	−0.083
	(−11.137) **	(−5.107) **	(−12.926) **	(−14.393) **	(−9.343) **	(−11.167) **
Percent of adults that report BMI ≥ 30	−0.091	−0.071	−0.039	−0.049	−0.042	−0.066
	(−7.985) **	(−4.558) **	(−4.295) **	(−5.063) **	(−4.274) *	(−6.826) **
Mentally unhealthy days per month	0.092	0.125	0.075	0.133	0.051	0.139
	(2.505) *	(2.28) *	(2.914) **	(5.321) **	(2.09) **	(5.402) **
Age-adjusted years of potential life lost rate	0	0.000	0.000	0.000	0.000	0.000
	(13.171) **	(9.648) **	(21.889) **	(13.398) **	(19.507) **	(22.248) **
Percent of White	−0.024	−0.019	−0.042	−0.051	−0.054	−0.048
	(−6.914) **	(−3.98) **	(−16.492) **	(−20.026) **	(−20.41) **	(−19.113) **
Percent of Native Americans	−0.058	−0.027	−0.116	−0.139	−0.136	−0.122
	(−8.285) **	(−2.837) **	(−19.505) **	(−21.984) **	(−22.855) **	(−23.033) **
Percent of Hawaiian or Pacific Islander	−2.274	−2.265	−1.929	−1.831	−1.943	−2.008
	(−5.37) **	(−3.715) **	(−4.361) **	(−3.93) **	(−4.109) **	(−4.957) **
Percent of Diabetic receiving HbA1c test	0.008	0.021	−0.018	0.024		
	(3.396) **	(7.894) **	(−3.294) **	(−4.156) **		
Crude motor-vehicle related mortality rate	−0.025	−0.031	−0.007	0.016		−0.019
	(−7.981) **	(−7.01) **	(−2.356) *	(4.443) **		(−5.508) **
Percent of single-parent households			0.022	0.030	0.032	0.026
			(5.042) **	(6.165) **	(6.544) **	(4.154) **
Chlamydia (STD) rate			0.001	0.001	0.001	0.001
			(4.083) **	(3.194) **	(4.681) **	(5.143) **
Percent of multi race			−0.137	−0.110	−0.111	−0.104
			(−3.695) **	(−2.66) **	(−2.794) **	(−2.763) **
Percent of children living in poverty	−0.014	−0.020				−0.019
	(−2.447) *	(−2.427) *				(−3.147) **
Percent of black	0.053	0.067				
	(11.355) **	(10.718) **				
Physically unhealthy days per month	0.114	0.160				
	(3.319) **	(3.097) **				
Age-adjusted homicide rate	0.033	−0.037				
	(3.707) **	(−2.366) *				
Percent of other race	−0.05			−0.080		−0.058
	(−3.072) **			(−4.163) **		(−3.843) **
Percent of Hispanic	0.016			0.020		0.024
	(2.371) *			(2.901) **		(4.025) **
Freshman graduation rate	0.004					
	(2.271) *					
Percent ZIP Code with a healthy food outlet	−0.004					−0.003
	(−2.764) **					(−1.938) *
Days with unhealthy Fine particulate matter		−0.024				
		(−2.012) *				
Days with unhealthy ozone			−0.014			−0.013
			(−2.889) **			(−2.284) *
Percent of adult who smoke					0.012	
					(2.679) **	
Primary Care Physicians rate					0.003	
					(2.802) **	
Violent crimes rate					0.000	−0.001
					(−2.014) *	(−3.803) **

* *t*-statistic is statistically significant at *p <* 0.05; ** *t*-statistic is significant at *p <* 0.01; and upper and lower values represent the coefficient and *t*-statistic, respectively.

**Figure 2:**
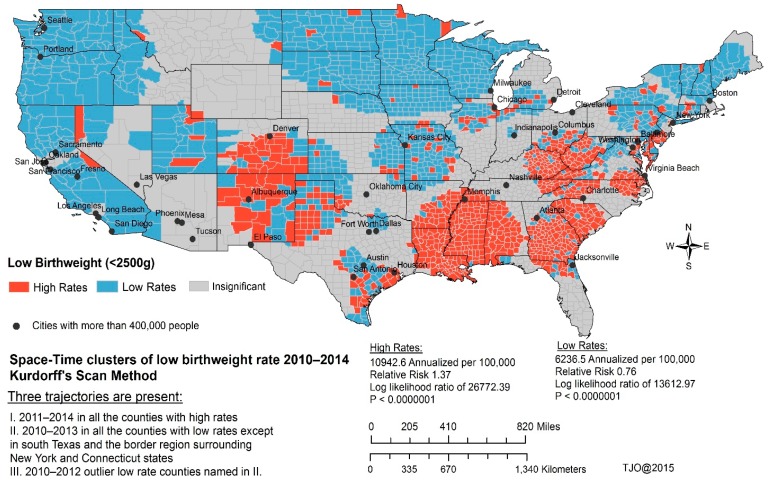
Spatiotemporal clusters of low birthweight for the period between 2010 and 2014.

Figure 2 shows spatiotemporal clusters of LBW over a five-year study period. The nine covariates for the first spatiotemporal model included: teen birth rate, percent of adults without insurance, percent of adults that report BMI ≥ 30, mentally unhealthy days per month, percent of diabetic Medicare enrollees receiving HbA1c test, days with unhealthy fine particulate matter, days with unhealthy ozone, percent of adults who smoke, and percent of African American population. The five covariates for the second spatiotemporal model included teen birth rate, percent of adults without insurance, percent of adults that report BMI ≥ 30, mentally unhealthy days per month, and percent of adults who smoke. The higher and lower than expected LBW rates in the first model incorporate two environmental quality variables that were found to be statistically significant.

In the first spatiotemporal model, we identified one statistically significant spatiotemporal cluster of LBW that was located in Henry County, Alabama. In the second spatial model, we identified two statistically significant spatiotemporal clusters of LBW that were located in Greene and Henry Counties, Alabama. Henry County was detected by both models. In the two models, it was evident that LBW outcomes appear to be partly explained by these covariates, which further lend support to the interaction and complexity of social, behavioral, and environmental factors.

### Summary of the Most Influential Predictors Presented in Table 2. The Predictors Explain Low Birthweight Outcomes and Were Consistently Present in the Following Time Points.

- Predictive variables present in all five time points: Ambulatory care sensitive conditions discharge rate, Teen births rate, Percent of adults 18–64 without insurance, Percent of adults that report BMI ≥ 30, Mentally unhealthy days per month, Age-adjusted years of potential life lost rate, Percent of white, Percent of Native Americans, and Percent of Hawaiian or Pacific Islander. Each of this variable was statistically significant at each time point.
- Predictive variables present in four of the time points: Percent of Diabetic receiving HbA1c test and Crude motor-vehicle related mortality rate.
- Predictive variables present in three of the time points: Percent of single-parent households, Chlamydia (STD) rate, and Percent of multi race.
- Predictive variables present in two of the time points: Percent of children living in poverty, Percent of black, Percent of other race, Percent of Hispanic, physically unhealthy days per month, and Age-adjusted homicide rate.
- Predictive variables only present in one time point: Freshman graduation rate, Percent ZIP Code with a healthy food outlet, Days with unhealthy Fine particulate matter, Days with unhealthy ozone, Percent of adult who smoke, Primary Care Physicians rate, and Violent crimes rate.

Thirty-seven percent of the counties in the spatiotemporal model had annualized low rates of birthweights of 6237 per 100,000 live births (relative risk 0.76, log likelihood 13,612.97, *p <* 0.0000001) while 28 percent of the counties had annualized high rates of birthweight of 10,943 per 100,000 live births (relative risk 1.37, log likelihood 26,772.39, *p <* 0.0000001). The remaining 35% of the counties in the model were statistically insignificant. Overall, the main areas of concern with elevated risk of LBW were identified in the southern and mid-western regions of the United States, it is especially pronounced in the counties of Mississippi, Alabama, Louisiana, Georgia, Arkansas, Tennessee, South Carolina, North Carolina, Kentucky, West Virginia, Ohio, Virginia, New Mexico, Texas, and Colorado. Demographically, the racial/ethnicity composition of counties with elevated LBW risk comprises 65% white, 17% African American, 10% Hispanics, and 8% others. However, these counties also have the largest proportion of African Americans (*i.e.*, about 67% of the total population of African Americans in the 48 contiguous state).

Furthermore, we identified three trajectories of LBW outcomes during the spatiotemporal modeling process (Figure 2). The first trajectory was observed between 2011 and 2014, this trajectory was associated with higher rates of LBW outcomes and was present in all the counties; the second trajectory was observed between 2010 and 2013, this trajectory was associated with lower rates of LBW outcomes and was present in all the counties except south Texas and counties that lie at the intersection of New York and Connecticut state boundaries; and the last trajectory was a set of outliers observed between 2010 and 2012, this trajectory represented counties with lower rates of LBW outcomes. These were identified in counties in south Texas (mostly Hispanics) and counties lying at the boundaries of New York and Connecticut states.

## 4. Discussion

For this study, LBW was used as a proof of concept to apply GIS and spatial analysis, spatiotemporal models, and traditional statistical analytics to an external exposome. The county-level predictive measures of LBW offers new insights into spatiotemporal patterns relative to key contributory factors. The findings in this study provide further evidence and are consistent with previous studies that have identified socioeconomic [29,30,31,32,33,34,35], behavioral [36,37,38], and environmental factors [39,40,41,42,43]. By means of our external exposome approach, we observed that LBW rate was more strongly associated with 17 variables. We consistently observed the influence of nine predictors in all the five models (ambulatory care sensitive conditions discharge rate, teen births rate, percent of adults 18–64 without insurance, percent of adults that report BMI ≥ 30, mentally unhealthy days per month, age-adjusted years of potential life lost rate, percent of White, percent of Native Americans, and Percent of Hawaiian or Pacific Islander). In some of the years, there were notable variations suggesting the changing influence of some of the predictors. However, after accounting for place as an explanatory variable, we identified LBW associations with five socioeconomic variables (teen births rate, percent of adults 18–64 without insurance, percent of adults that report BMI ≥ 30, mentally unhealthy days per month, and percent of adults who smoke) in two counties (Greene and Henry Counties in Alabama); all the counties identified with higher than expected LBW rates were also significantly associated with two environmental quality variables (ozone and fine particular matter).

It was evident from the county-level health measures that the southern portion of the United States has consistently experienced poor pregnancy-related outcomes during the study period spanning from 2010 to 2014. The spatiotemporal models provide further evidence of LBW hotspots and are driven by a mixture of key socioeconomic, behavioral, and environmental factors. Furthermore, the profiles of two counties with statistically significant spatiotemporal clusters of LBW were consistent with prior Alabama’s Department of Public Health Annual Reports of 2010, 2011, 2012, and 2013. According to Alabama’s Health Disparities Status Report of 2010, the disparity has worsened from 82% higher in 2000 to 92% higher in 2008 for African Americans when compared to Whites. The racial/ethnicity composition of Greene County consists of 86% African Americans; while, the composition of Henry County is 31% African American. Both of the counties are situated in the rural part of Alabama, where many hospitals lack basic health care services such as obstetrical services (Alabama Department of Public Health Annual Report 2013). Greene County was ranked last in the state health report due to poor health indicators. In this report, Greene County’s low weight births increased from 9.4 percent in 2001 to 16.7 percent in 2011 (Alabama Department of Public Health Annual Report 2013).

Within the south, we recommend place-based targeted interventions to prevent and control pregnancy-related morbidities and mortalities with the target being low income, unmarried, African American women. We believe such intervention measures will have an effect of a set of socioeconomic, behavioral, and environmental factors and improve pregnancy-related health outcomes. In addition, we recommend efforts to prevent and control identified risk factors should be implemented before and during pregnancy, because there are likely to have an impact on perinatal health outcomes.

In spite of the key insights derived from this study, there are some limitations that must be recognized because there is no prefect model. First, some of the measures in this study are derived from either objective or subjective reports. Second, health behavior measures are not adjusted in the county ranking data. There are other concerns that are related to temporal misalignment; large units may mask differences within areas; and small numbers problem. In the future, as more accurate secondary longitudinal data becomes readily available, we expect to use it to refine our spatiotemporal models.

## 5. Conclusions and Implications

The causes of pregnancy-related morbidities and mortalities are numerous and include a broad array of factors from across multiple environmental domains. The development of a multi-level, multi-dimensional surveillance system is needed to increase our understanding of the broad array of risk and protective factors associated with pregnancy-related morbidities and mortalities.

The external exposome approach and tools provide a theoretically agnostic, data driven model that holds considerable promise as an alternative to traditional, discipline-specific, hypothesis-driven models for understanding health disparities. It supports both data-driven and hypothesis-driven approaches, and can be used both to generate and to test hypotheses. An external exposome approach offers new ways for conceptualizing the underlying causes of health disparities and the biological pathways through which environmental exposures affect human development and health. By incorporating spatial and temporal dimensionalities, an external exposome framework provides a data structure that accommodates a life span approach, a cumulative risk model, and extended periods between exposure and expression and detection of a disease. Furthermore, an external exposome approach is able to incorporate methods and analytics from various disciplines and can handle multiple types of data with varying spatial and temporal dimensions. This approach holds considerable promise in unraveling the complex nature of health outcomes and disparities data. The multi-dimensional nature of this approach can support a broad array of analytics including predictive modeling, simulation, Bayesian networks, and structural equation modeling, which can be used to produce network of hypotheses concerning factors that might be manipulated to improve pregnancy-related disparities.

An exposome framework has implications for identifying populations at greatest risk for health disparities and for increasing our understanding of the underlying molecular pathways through which environmental exposures affect human health and development and result in disparities at a population level. It holds great promise for the deployment of place-based, targeted interventions that are responsive to local risk factors that underlie protracted disparities in pregnancy-related morbidities and mortalities and other diseases. While this paper uses county level data to describe the link between health outcomes and external exposome, the approach can be readily scaled spatially and temporally to address other units of analysis (e.g., neighborhoods). Applying an external exposome approach can assist in developing and targeting interventions to those at greatest risk.

### Future Directions

This analysis will be applied to three other pregnancy-related [32] morbidities and mortalities (infant mortality rates, maternal mortality, and prematurity) in future studies. While data presented in this paper are cross-sectional in nature, we are currently working on methods to apply this general approach to the analysis of longitudinal data. In addition, we will continue to expand and harmonize additional datasets to incorporate into the current model. This line of research should help unlock the explanatory and predictive power of innovative methods such as those we have discussed here.
